# MicroRNA miR-27b-3p regulate microglial inflammation response and cell apoptosis by inhibiting A20 (TNF-α-induced protein 3)

**DOI:** 10.1080/21655979.2021.1969195

**Published:** 2021-12-13

**Authors:** Liping Li, Chao Qi, Yuanyuan Liu, Youliang Shen, Xia Zhao, Han Qin, Yi Zhang, Tengbo Yu

**Affiliations:** a Department of Orthopedic Surgery, The Affiliated Qingdao Central Hospital of Qingdao University, Qingdao, Shandong, China; b Department of Orthopedic Surgery, The Second Clinical Medical College of Qingdao University, Qingdao, Shandong, China; c Department of Orthopedic Surgery, Affiliated Hospital of Qingdao University, Qingdao, Shandong, China; d Department of Oncology, The Affiliated Qingdao Central Hospital of Qingdao University, Qingdao, Shandong, China; e Department of Oncology, The Second Clinical Medical College of Qingdao University, Qingdao, Shandong, China

**Keywords:** miR-27b-3p, A20, microglia, inflammation, spinal cord injury, cerebral hemorrhage

## Abstract

Inflammatory reaction exerts a pivotal role in secondary damage after cerebral hemorrhage and spinal cord injury. miRNAs can both promote and inhibit inflammatory actions among microglial cells. The objective of the present paper was to figure out whether miR-27b-3p produced regulatory effects during processes of microglial inflammation. Lipopolysaccharides (LPS) were used to prepare microglial activation models. Following miR-27b-3p overexpression and interference, the RNA and protein levels of tumor necrosis factor (TNF)-α, interleukin (IL)-6, and IL-1β were subjected to real-time fluorescent quantitative PCR (qPCR) and western blot assays, respectively. Cellular apoptosis was subjected to flow cytometry and miR-27b-3p target genes were visualized using a dual luciferase reporter system for verification. The levels of TNF-α, IL-6, and IL-1β mRNA in miR-27b-3p-overexpressed microglial cells were markedly increased compared to the control. Apoptosis of microglial cells was increased markedly in the overexpressed miR-27b-3p group compared to the negative control. Conversely, a different result was presented in the microglial transfected with miR-27b-3p inhibitors. The downregulation of A20, a miR-27b-3p target gene, mediated levels of TNF-α, IL-6, and IL-1β. Furthermore, A20 reduced microglial apoptosis. These data revealed that miR-27b-3p could mediate not only microglia activation but also neuroinflammation via downregulating A20 expression. Thus, miR-27b-3p is regarded as gene therapy in treating cerebral hemorrhage and spinal cord injury.

## Graphical abstract

**Figure:**
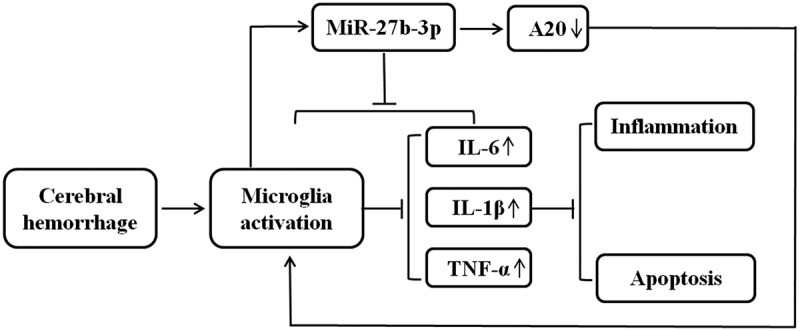

## Introduction

Cerebral hemorrhage and spinal cord injury is recognized as a refractory disease that frequently arises in neurosurgery with high rates of morbidity, mortality, and disability. Subarachnoid hemorrhage and intracerebral hemorrhage are both categorized as cerebral hemorrhage and spinal cord injury because the occurrence of pathologic processes for both primary and secondary brain injuries are identical[1], [2]. Primary injuries are the consequence of hematoma growth. It develops after the onset of stroke within the first few hours and induces injuries to the surrounding tissues and neurons [3]. Secondary brain injuries are a series of pathologic alterations induced by hematoma thereby resulting in multiple neurologic aggravations in activating cytotoxicity, excitotoxicity, oxidation, and inflammatory response [4]. Importantly, the inflammatory response acts as a crucial role in secondary injury after cerebral hemorrhage and spinal cord injury [5]. Microglia represents the most important inflammatory effector cell in the central nervous system (CNS), taking up approximately 15% of the total cerebral glial cells. Meanwhile, it can be activated immediately even in minutes when cerebral hemorrhage occurs [6]. Following microglia activation, multiple pro-inflammatory cytokines and chemokines were released facilitating the accumulation of inflammatory cells in the course of cerebral hemorrhage [7,8]. Typically, the double phenotypes M1 and M2 were the polarized microglia and characterized by features of both inflammation and anti-inflammation. The M2 phenotype contributes to tissue repair and anti-inflammatory cytokine production [9]. Inflammatory M1 phenotype can be induced by LPS stimulation and acts as inflammation promoting cytokines namely IL-1β, TNF-α, and IL-6 [10]. Iba1 (Ionized calcium binding adapter molecule 1) are the markers of activated microglia mediated by inflammation [10,11].

MicroRNAs are categorized into small endogenous non-coding RNAs, containing 18–25 base pairs. By binding specifically to the target genes at the 3’UTR (untranslated region) area, they can downregulate the expression of these genes. Guo et al compared the expression of peripheral blood miRNAs amongst spontaneous cerebral hemorrhage, ischemic stroke, and normal people [12,13] and they found that there were 70 miRNAs differentially expressed in male subjects (48 upregulated, 22 downregulated), and 42 microRNAs differentially expressed in females (37 upregulated and 5 downregulated). miRNAs are critical regulators for microglia and they potentially exert anti- or pro-inflammatory actions [14,15]. For example, as an inflammatory promotor, miR-155 worsens the inflammation of microglia while miR-146a acts as an inflammatory inhibitor that is capable of reducing inflammation [16,17]. miR-331-3p has been reported in a recent study that it is responsible for downregulating the expression of TNF-α and IL-6, thereby alleviating the protective reactions of the injured tissues and benefiting in promoting the neurological function recovery in mice following the episode of intracerebral hemorrhage [18]. A previous study indicated that miR-27 might be related to CNS dysfunction [19]. MiR-27a, a member of the miR-27 family, highly expressed in endothelial cells, is present in the central nervous system and controls cellular apoptosis [20]. Furthermore, the study has shown that miR-27-3p can promote microglia activation and inflammation-related factor expressions in rats [21].

Zinc finger protein A20 is recognized as potent tumor necrosis factor-alpha-induced protein 3, known as TNFAIP3, and it is characterized by inhibiting the activity of tumors. The human A20 gene is located in the region of chromosome band 6q23.3, containing 790 amino acids and the molecular weight is measured at 90 kDa [22]. A20 protein is hardly expressed under normal body circumstances; however, a high level of A20 protein can be observed in almost all of the cells under pathological environment: trauma, infection, and stress [23]. An inducible A20 expression *in vivo* contributes to the attenuationing of inflammatory response and alleviation of damage caused by excessive inflammatory reaction [24], which is one of the important endogenous anti-inflammatory protective mechanisms of the body. Studies have shown that high expression of A20 helps to reduce the inflammation mediated by TNF-α, LPS, FAS, and toll-like receptors. However, the knockout of A20 *in vivo* makes for a systemic inflammatory response resulting in the premature death of mice at an 8-week survival rate only about 40% [25,26,27]. Additionally, the knockout of A20 *in vitro* activated the microglia cells allowing to release plenty of inflammation promotors and chemokines TNF-α, IL-6, and IL-1, enhancing the spontaneous inflammatory response of neurons [22]. The crucial role of A20 in regulating microglia activation during CNS homeostasis and pathology has been demonstrated, indicating an essential role of A20 in the control of microglia activation and neuroinflammation [24].

Although the role of A20 in microglia activation has been investigated, the upstream regulatory genes of A20 are poorly understood [27, 28]. It has been demonstrated that A20 and miR-27 play a role in microglia activation and neuroinflammation. And in our previous study, miR-27b-3p was found to be related to A20 expression. Therefore, the purpose of this research seeks to figure out what role of miR-27b-3p produces in microglia activation and neuroinflammation. In the present study, we sought to explore the role of miR-27b-3p in regulating microglial inflammatory response, validate its role of being an upstream regulatory gene of A20, and provide a novel research basis for gene therapies in treating cerebral hemorrhage and spinal cord injury.

## Materials and methods

### Animals and materials

Thirty newborn (2–4 d) and specified pathogen-free male C57BL/6 mice were collected from Pengyue company (Shandong, Jinan) [29]. The animal experiments were supported by the Affiliated Hospital Ethics Committee of Qingdao University.

### Isolation, culture, and purification of mice primary microglia

Primary microglia culture was derived from the whole brain cortex of mice early after their birth (P0) as described [30]. Under sterile conditions, the newborn mice were soaked in 75% ethanol for disinfection and then sacrificed by decapitation. Following the brains were removed through craniotomy, the separated vessels and mucous membranes were washed with D-Hanks solution (Solarbio, China). Vascular membrane tissues were prepared and sliced into approximately 1 mm^3^ in thickness, subsequently digested using 2.5% trypsin (HyClone, USA) under the temperature of 37°C for 10 min and terminated using a mixture of complete DMEM/F12 (HyClone, USA) medium following enzymatic reactions. The mixture was prepared with 15% FBS (Fetal Bovine Serum), 100 U/mL Penicillin, and 0.4 mg/mL DNAse and then incubation was conducted under a 37°C condition for 10 min. Through a 200 mesh cell sieve, the filtered liquid was collected and centrifugated at 500 g for 5 min. Following the supernatant was discarded, a complete medium was supplemented for cell resuspension. After that, the cells were seeded in a 75 cm^2^ culture flask and cultured in a humidified 37°C incubator containing 5% CO_2_ for 5 d. The solution was refreshed every 3 d. Following additional 9–14 d, the primary microglial cells were shaken off from the astrocytic layer and harvested due to different adhesion properties. After treatment with 100 ng/mL LPS for 24 h, the microglial cells were subsequently inoculated in plates of different wells at an appropriate density for further experiments.

### Immunofluorescence detection

The obtained primary microglial cells derived from mice were seeded in 24-well plates and lasted for 24 h. Partial cells were processed with 100 ng/mL LPS, and the remaining untreated cells were served as negative control. Cell slides were prepared and the microglial cells were subsequently treated with a 4% paraformaldehyde fixation agent for 15 min. PBS (phosphate buffered saline) was used to wash 3 times, 3 min each time, and these sections were added with 5% BSA (Bovine Serum Albumin) for immersion for 30 min. After that, the blocking agent was aspirated, and Iba1antibody (1:200, ab178847, Abcam, UK) was added for incubation at 4°C overnight. Subsequently, PBST (0.05% Tween-20) was employed to wash the slides clean and repeated 3 cycles of washing, 3 min each time. Following the addition of goat anti-rat IgG H&L, secondary antibodies at 1:1 000 (ab150077, Abcam, UK), the incubation was carried out at 37°C for 1 h. After rinsing three times with PBST, 3 min each time, DAPI was added, and incubated 10 min in the dark followed by 3 cycles of PBST rinsing, 5 min each time [31]. The cells were ultimately visualized under a fluorescence microscope (Olympus, Japan). Red represented positive cells. A20 expression was detected using the A20 antibody (1:200, ab13597, Abcam, UK).

### ELISA assay

Microglia were treated with LPS (Sigma, USA) at 100 ng/mL wtih three set time-points (1-, 3-, and 7-d) to identify inflammatory responses, untreated microglia cells were served as negative controls. The concentration of inflammatory factors TNF-α (ab208348), IL-1β (ab197742), and IL-6 (ab203360) was determined following the instructions of use of the ELISA kit (Abcam, UK). The experimental procedures were carried out following instructions. Detection of optical density for each well was performed at 450 nm wavelength.

### Cell transfection and vector construction

Following 24 h treatment of the microglial cells with 100 ng/mL LPS, we conducted vector construction and transfection in mouse microglial cells [32]. The miR-27b-3p mimics, lentiviral recombinant vector LV-A20 overexpression, LV-siRNA-A20, miR-27b-3p inhibitor, as well as relevant negative controls (NC) were all sourced (GenePharma, China). The sequences were as follows: miR-27b-3p mimic, 5’-TTCACAGTGGCTAAGTTCTGC-3’; miR-27b-3p inhibitor, 5’-GCAGAACTTAGCCACTGTGAA-3’; miR-NC, 5’-GGTTCCATCGTACACTGTTCA-3’ and anti-miR-NC, 5’-CCATCAGTCCCCATCGCCA-3’. They were transfected at the concentration of 100 μM into the microglial cells with Lipofectamine 2000 (Invitrogen, USA). The microglial cells were initially implanted into 6-well plates, at a density of 2 × 10^5^/mL, transfected at 70% confluency, and incubated subsequently under the condition of 37°C with 5% CO_2_ for 48 h for further analysis. Primer sequences used were presented (refer to Supplementary Table S1).

### Flow cytometry to detect microglial cell apoptosis

The detection of microglial cell apoptosis was performed using the Annexin V-FITC Apoptosis Detection Kit I (BD, USA), and the procedures were performed under the guidance of the instructions of use [33]. LPS- and transfected- treated microglia samples were collected by centrifugation at 300 g for 5 min and subsequently washed twice at the temperature of 4°C with pre-cooled PBS. To the cells, 250 μL binding solution was supplemented for resuspension, and the diluted rate was prepared at 1:4 with deionized water at room temperature. The concentration of the medium was adjusted at 1 × 10^6^ cells/mL. After that, a 5 mL flow cytometry tube was used to assist in transfecting 100 μL cell suspension, followed by incubation with the addition of 15 μL PI and 5 μL Annexin-V-FITC. The process was carried out avoiding light at room temperature for 15 min. The ultimate assessment of apoptosis was conducted via FACSCalibur (BD, USA).

### Real-time PCR

Firstly, we extracted total RNA from the microglial cells according to the procedures of TRIzol reagent (Invitrogen). Then we synthesized the first strand cDNA to start the assay with gDNA Eraser (Takara, Japan) by a PrimeScript™ RT reagent kit. qRT-PCR was subsequently conducted through a real-time PCR system (Bio-rad, USA) using the reagent of SYBR Green PCR Kit (Takara, Otsu, Japan). Reaction procedures of qRT-PCR were performed based upon the instructions of use. Thermocycling protocol was designed and described as the following three steps: 95°C for 30 sec, 40 cycles of 95°C for 5 sec, 60°C for 30 sec and 72°C for 15 sec. By adopting the 2^−ΔΔCT^ method, the relative expression levels were calculated. Expressions in cardiomyocytes were quantified as well and relevant expression levels were normalized to β-actin or small non-coding RNA U6. Relevant primer sequences utilized during the PCR detection were presented (as shown in Supplementary Table S2).

### Western blot to detect protein expression*[34]*

The microglial cells were initially lysed using RIPA reagent (Beyotime, China). Then the concentration of the extracted protein was quantified using BCA assays (Takara Biotechnology Co., Ltd.). SDS-PAGE gel at 10% was prepared, 40 μg of total protein was added to each well for the following electrophoresis running at 120 V for 2 h. When the electrophoresis was completed, the used PAGE gel was discarded. The protein was transferred to a PVDF membrane under a wet condition due to its less tendency to failure than the semi-dry transferrence and poured an appropriate quantity of 5% BSA blocking buffer (Solarbio, China) to block the membrane for approximately 1 h at room temperature. The PDVF membrane was rinsed with TBST (0.05% Tween-20) 3 times for follow-up experiments. Primary antibodies (1:500) was supplemented and incubated with the PDVF membrane for 2 h at room temperature, and followed by 3 times of TBST rinsing for 5 min, added with secondary antibody (1:1 500, ab205718, Abcam), incubated with the PDVF membrane for 2 h at room temperature, and finally rinsed with TBST three times for 5 min. Protein visualization was conducted using an enhanced chemiluminescence detection kit (Solarbio, China). Densitometry was subsequently semi-quantified with an ImageJ 1.8.0 software (National Institutes of Health). The experimental primary antibodies included anti-IL-1β (ab2105, Abcam), anti-IL-6 (ab7737, Abcam), anti-TNF-α (ab66579, Abcam), Anti-TNFAIP3 (ab92324, Abcam) and anti-β-actin (ab8227, Abcam).

### Dual luciferase reporting system

Briefly, 293 T cells were planted into 96-well plates and adjusted at a density of 1 × 10^4^ cells. miRNA-27b-3p sequence and the binding sites in the 3’UTR region of target genes were predicted using related platforms including miRBase, Targetscan 7.2, PicT AR WEB INTERFACE, and TargetScanFish 6.2. Either the miR-27b-3p mimic sequence or negative control sequence and dual luciferase vector (psiCHECK-2) with either A20 wild-type (WT) sequence or mutant (MUT) sequence were cotransfected using Lipofectamine 2000 (Invitrogen). The restriction site was at Xho I and Not I. The sequences included A20 WT, 5’-CUGUGAA-3’, and A20 MUT, 5’-GACACUU-3’. The Dual-Glo® Luciferase Assay System (Promega, USA) was employed to detected luciferase activities of cells based upon the instructions of use and normalized subsequently based upon the signal ratio of Renilla-to-firefly luciferase.

### Statistical analysis

Statistical data were collected from the results of at least three independent experiments and presented as means ± SD. The SPSS 17.0 software (SPSS, Chicago, IL, USA) was employed for the analysis of collected data. Pairwise comparison was analyzed by utilizing two-tailed Student’s t-tests and data comparison among three or more groups adopted the one-way analysis of variance (ANOVA) with Dunnett’s tests. Findings were considered statistically significant at a p-value of <0.05.

## Results

**Figure 1.**
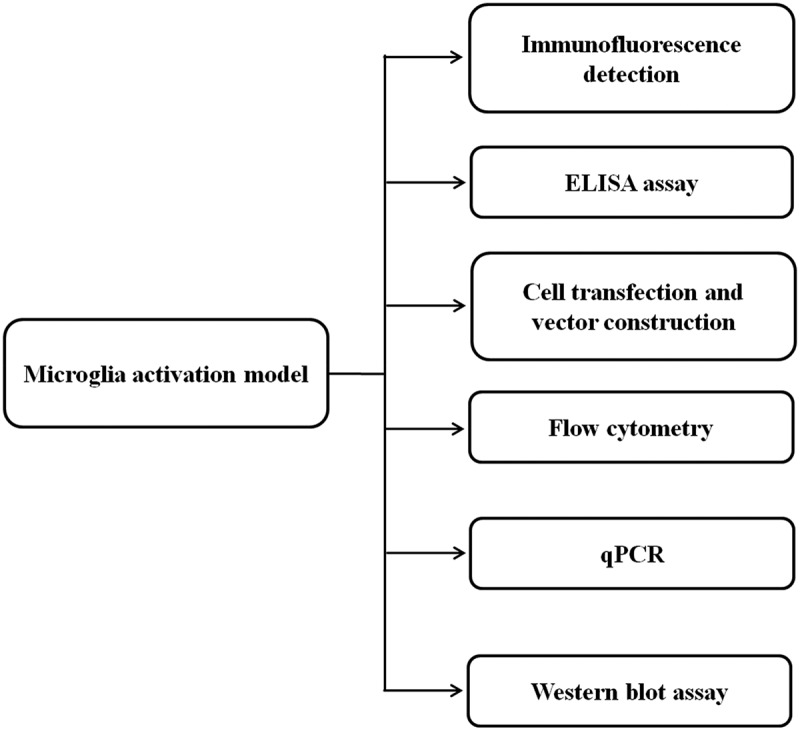
An overall flowchart for the research methodology

We firstly used LPS to construct a microglia activation model, and induced neuroinflammation through this model. Iba1 was selected as a microglia activation marker. Through the established model, qPCR, ELISA, and Western blot assays were conducted to verify how miR-27b-3p affected the functions of microglia and regulated the inflammatory response of microglia. An overall flowchart of research work methodology as shown in Figure 1.

### Microglia isolation and construction of an inflammation model

**Figure 2.**
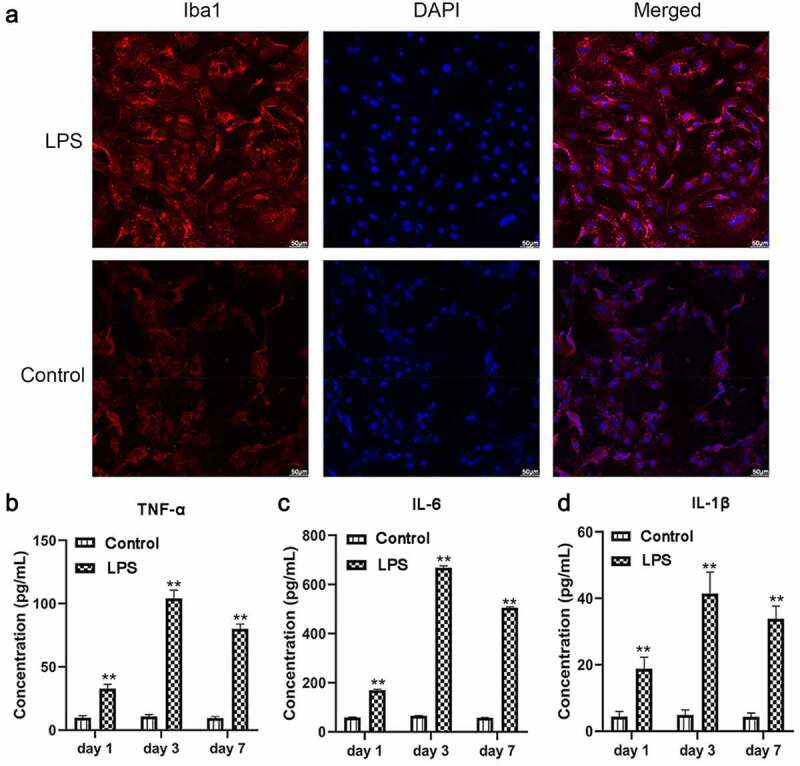
After 100 ng/mL LPS treatment on microglia, protein expressions of inflammatory factors and microglial cell marker Iba1 were determined (n = 3). (a) Immunofluorescence detection of Iba1 expression. (b-d) ELISA detection of TNF-α, IL-6, and IL-1β expression. The scale bar is 50 μm. **, P < 0.01 compared with the control

We firstly constructed a microglial activation model using LPS, through which neuroinflammation and expression of inflammation promoters were induced [24]. Iba1 as an activated microglia marker was highly expressed following LPS stimulation (Figure 2a). Additionally, ELISA assays indicated that TNF-α, IL-6, and IL-1β levels were higher in LSP-treated microglia than in the control with P < 0.01 (Figure 2b-2d). These findings indicated that the microglial cells were successfully isolated in this study and the inflammation model of microglial cells was successfully constructed.

### miR-27b-3p induces a microglial inflammatory response

**Figure 3.**
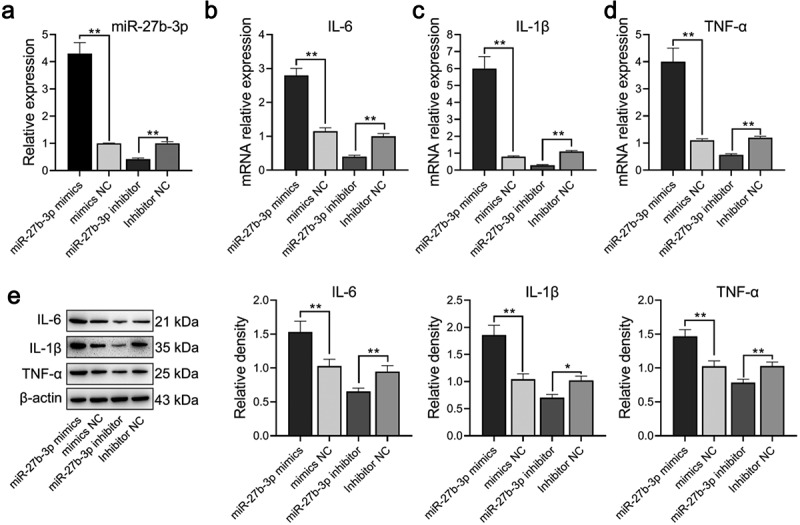
miR-27b-3p promoted expressions of TNF-α, IL-6, and IL-1β in microglial cells treated with 100 ng/mL LPS (n = 3). (a) Expression of miR-27b-3p determined in transfected microglial cells. (b-d) TNF-α, IL-6, and IL-1β mRNA expression were determined by qPCR. (e) Western blot assay was used for detecting TNF-α, IL-6, and IL-1β protein expressions. The relative density was measured by Image J. mimic-NC: mimic negative control; si-NC: inhibitor negative control. *, p < 0.05; **, p < 0.01 compared to control

**Figure 4.**
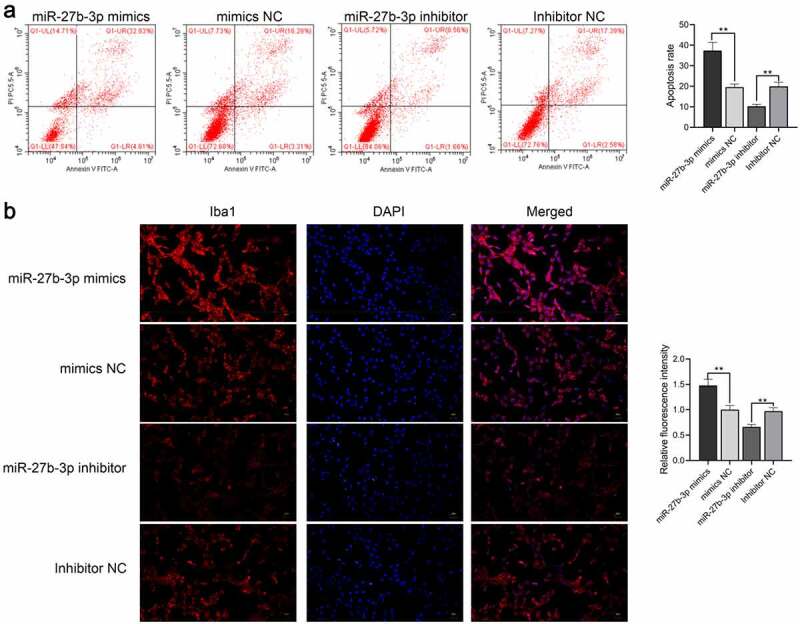
miR-27b-3p accelerated apoptosis and Iba1 expression inmicroglial cells treated with 100 ng/mL LPS (n = 3). (a) Flow cytometry for detecting apoptosis of miR-27b-3p in microglia. (b) Immunofluorescence for detecting Iba1 expression. **, P < 0.01 compared with the NC control

Through the construction of the neuroinflammation model, we verified whether miR-27b-3p influenced the function of microglia, LPS-treated microglial cells were transfected with either overexpressed or interfered miR-27b-3p. Analysis of qPCR assay results indicated successful overexpression or silence of miR-27b-3p in the microglial cells with P < 0.01 (Figure 3a). qPCR and Western blot findings illustrated that the expression of TNF-α was considerably elevated at mRNA and protein levels in the miR-27b-3p mimic whereas dramatically declined in the miR-27b-3p inhibitor (Figure 3b-e). The possible role of miR-27b-3p in microglia apoptosis was identified using flow cytometry assays. Apoptosis of microglial cells in the miR-27b-3p mimics, the mimic-NC, the miR-27b inhibitor, and the inhibitor NC group were 37.44%, 19.59%, 10.22%, and 19.97%, respectively (Figure 4a). Compared with the negative control, immunofluorescence revealed that Iba1 was highly expressed in the miR-27b-3p mimics group whereas it was poorly expressed in the miR-27b inhibitor group (Figure 4b). In general, miR-27b-3p promoted microglial inflammation and accelerated its apoptosis.

### miR-27b-3p target A20 gene

**Figure 5.**
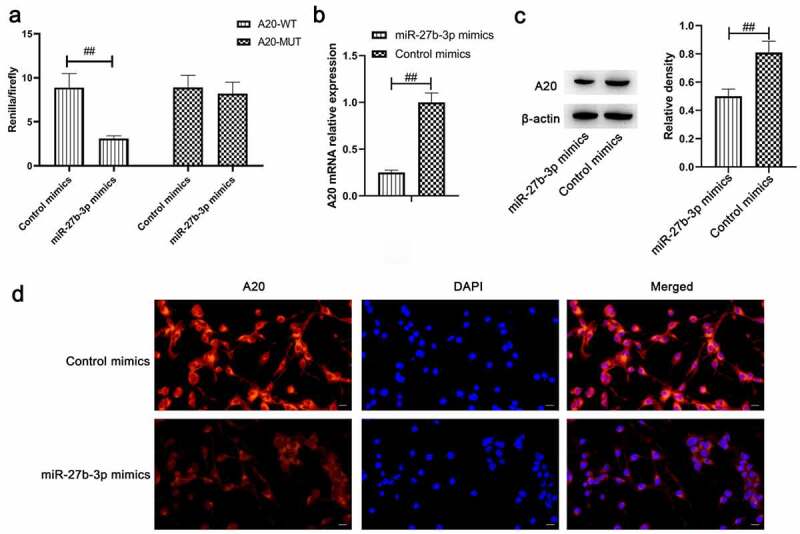
A20 target gene of miR-27b-3p (n = 3). (a) Overexpressed miR-129-5p downregulated the luciferase activity of only wild-type A20 rather than mutant A20 in 293 T cells. (b) qPCR detection on A20 following miR-27b-3p overexpression in microglial cells. (c) Western blot assay was employed to determine A20 expression following overexpressed miR-27b-3p in microglial cells. (d) Immunofluorescence detection of A20 in microglial cells by overexpressed miR-27b-3p. The scale bar is 20 μm. ##, p < 0.01 compared with the Control mimics

Upon database comparison in the StarBase and literature review, all findings indicated A20 as a potential target of miR-27b-3p. Meanwhile, it plays a crucial role in the regulation of microglial inflammation, which further underpinned our hypothesis [35]. Accordingly, we initially conducted a dual luciferase assay to assess the hypothesis that A20 might be a target gene of miR-27b-3p. Based on this hypothesis, pmirGLO-A20-3’UTR and pmirGLO-A20-3’UTR-mutation vectors were construed, the validation assay was conducted in 293 cells. The results suggested that the fluorescence activity of pmirGLO-A20-3’UTR and miR-27b-3p in the co-transfection group was reduced, whereas there was no change in pmirGLO-A20-3’UTR-mutation (Figure 5a). Besides, overexpressed miR-27b-3p remarkably reduced A20 expression and that was deeply verified in the results of qPCR, Western blot, and immunofluorescence (Figure 5b-d). Taken together, all the previously illustrated findings indicated that A20 was the target gene of miR-27b-3p.

### A20 inhibits microglial inflammation

**Figure 6.**
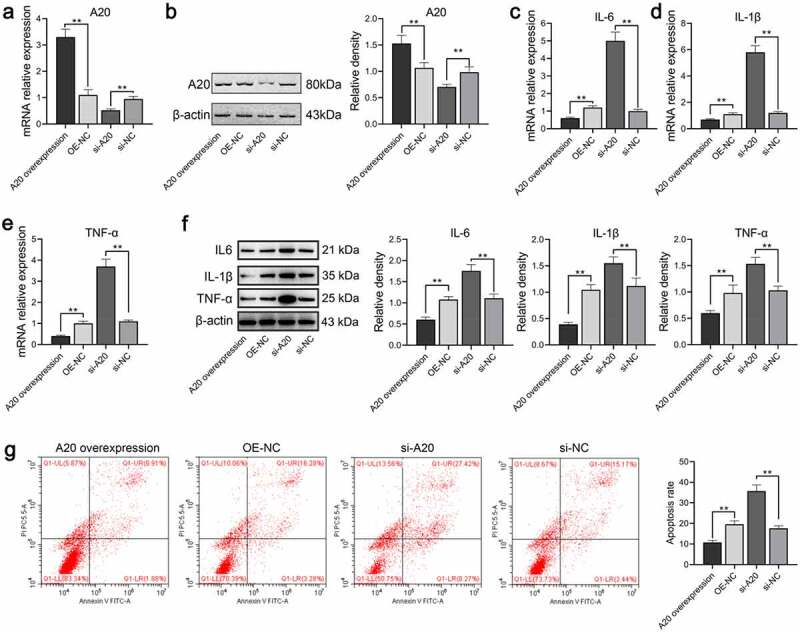
A20 inhibited TNF-α, IL-6, and IL-1β expressions in microglial cells treated with 100 ng/mL LPS (n = 3). Both qPCR assay (a) and western blot assay (b) were adopted for the detection of A20 mRNA and protein expression, respectively. (c-e) mRNA levels of TNF-α, IL-6, and IL-β were subjected to qPCR. (f) Protein levels of TNF-α, IL-6, and IL-β were subjected to western blot. The relative density of TNF-α, IL-6, IL-1β, and A20 proteins was measured by Image J. (g) Apoptosis of microglia was detected by flow cytometry. OE-NC: overexpression negative control; si-NC: siRNA negative control. **, p < 0.01 compared with the negative control

Following the verification of qPCR and Western blot assays, A20 was successfully overexpressed or silenced in microglial cells compared with corresponding negative control with P < 0.01 (Figure 6a, b). The mRNA and protein levels of TNF-α, IL-6, and IL-1β were markedly increased in the si-A20 whereas greatly decreased in the A20 overexpressed cells (Figure 6c-f). The effects of A20 on microglial apoptosis were determined via flow cytometry which suggested that apoptosis of A20 overexpressed, the NC, the si-A20, and the si-NC were 10.79%, 19.56%, 35.69%, and 17.61%, respectively (Figure 6g) which revealed that A20 inhibited microglial inflammation and apoptosis.

## Discussion

Microglial cells represent the intrinsic macrophage response of the CNS. The first several effectors in the CNS can be impaired under diseased conditions where the early pro-inflammatory factors’ release depends on neutrophils, monocytes, and lymphocyte-aggregated microglia, of which the microglial cells are the nutritional and supportive players in brain tissues [36]. The consequence of excessively activated microglia can lead to neuronal synaptic injury and neuronal function impairment, which participate in multiple reactions associated with neuroinflammation and impinge on the pathological process of diseases [37].

Several studies have demonstrated when microglial cells are activated, they are responsible for secreting IL-6, TNF-α, and IL-1β to promote neuroinflammation [38–40]. The present study observed microglia activation and neuroinflammation in LPS-treated microglia. Following LPS stimulation of microglia, we observed an increase in the Iba1 expression. At the same time, the secretion levels of inflammation promoting cytokines including IL-6, IL-1β, and TNF-α were also elevated to a certain extent. Some researchers have reported A20 protein is associated with the adaptive immune response process of the human body to achieve its regulatory function, and thus modulates the production of inflammatory factors or transmitters and improves the pathological process of inflammatory diseases [22,24]. A20 is essential in controlling the activation of microglial cells whenever it is under the condition of a steady state or a neuroinflammatory state. Moreover, when the Nlrp3 inflammasome is under an overactivated condition, the absence of microglia A20 worsens several sclerosis-like disorders and upregulates the secretion of IL-1β cytokine [24]. Our results indicated that A20 produced a role in inhibiting IL-6, IL-1β, and TNF-α expression in microglial cells. Furthermore, A20 positively affected the microglial cells by reducing apoptosis. Numerous evidence has shown that miRNAs regulate microglia activation and participate in the associated inflammatory response [8,12,25]. This study presented our findings that miR-27b-3p was responsible for improving the expression of IL-6, IL-1β, and TNF-α cytokines. Meanwhile, it is also attributable to the promotion of microglial apoptosis. A severe consequence of nerve depolarization and glutamate release is the induced neuronal death, which is caused by highly expressed pro-inflammatory cytokines in microglia [41]. As the process of cell death develops, series of changes are accompanied accordingly including swollen cells and ruptured plasma membranes. As a result, these alternations are responsible for a great deal of cytosolic content leakage and various inflammatory reactions [24]. We also verified that miR-27b-3p overexpression was attributable to the inhibition of A20 expression. Then, we speculated that miR-27b-3p could negatively influence microglial cells which might be realized by reducing the expression of A20.

## Conclusion

Taken together, miR-27b-3p could induce microglial activation, promote the increase of IL-6, IL-1β, and TNF-α expression, and promote cell apoptosis. Our experiments demonstrated that overexpression of miR-27b-3P would inhibit A20 activation which implied that A20 was the target gene of miR-27b-3p. It might increase the expression of inflammatory factors and promote microglial cell apoptosis by regulating A20, thus leading to the aggravation of neuroinflammation in microglial cells.

## Supplementary Material

Supplemental Material

## Highlights

- miR-27b-3p increased the apoptosis rate of microglial cells.
- miR-27b-3p increased the levels of TNF-α, IL-6, and IL-1β expression.
- A20 is the target gene of miR-27b-3p.

## References

[1] Ye L, Gao L, Cheng H. Inflammatory profiles of the interleukin family and network in cerebral hemorrhage. Cell Mol Neurobiol. 2018;38:1321–1333. 30027390 10.1007/s10571-018-0601-x PMC11481843

[2] Chen B, Wang H, Lv C, et al. Long non-coding RNA H19 protects against intracerebral hemorrhage injuries via regulating microRNA-106b-5p/acyl-CoA synthetase long chain family member 4 axis. Bioengineered. 2021;12:4004–4015. 34288826 10.1080/21655979.2021.1951070 PMC8806815

[3] Keep RF, Hua Y, Xi G. Intracerebral haemorrhage: mechanisms of injury and therapeutic targets. Lancet Neurol. 2012;11:720–731. 22698888 10.1016/S1474-4422(12)70104-7 PMC3884550

[4] Zhu H, Wang Z, Yu J, et al. Role and mechanisms of cytokines in the secondary brain injury after intracerebral hemorrhage. Prog Neurobiol. 2019;178:101610. 30923023 10.1016/j.pneurobio.2019.03.003

[5] Jian YP, Dong SJ, Xu SS, et al. MicroRNA-34a suppresses neuronal apoptosis and alleviates microglia inflammation by negatively targeting the Notch pathway in spinal cord injury. Eur Rev Med Pharmacol Sci. 2020;24:1420–1427. 32096191 10.26355/eurrev_202002_20199

[6] Yang K, Wang Z. Rab18 interacted with V-set and immunoglobulin domain-containing 4 (VSIG4) to involve in the apoptosis of glioma and the sensitivity to temozolomide. Bioengineered. 2021;12:1391–1402. 33904378 10.1080/21655979.2021.1919012 PMC8806276

[7] Jin Y, Yao G, Wang Y, et al. MiR-30c-5p mediates inflammatory responses and promotes microglia survival by targeting eIF2α during Cryptococcus neoformans infection. Microb Pathog. 2020;141:103959. 31958475 10.1016/j.micpath.2019.103959

[8] Karali M, Guadagnino I, Marrocco E, et al. AAV-miR-204 protects from retinal degeneration by attenuation of microglia activation and photoreceptor cell death. Mol Ther Nucleic Acids. 2020;19:144–156. 31837604 10.1016/j.omtn.2019.11.005 PMC6920266

[9] Ma Y, Wang J, Wang Y, et al. The biphasic function of microglia in ischemic stroke. Prog Neurobiol. 2017;157:247–272. 26851161 10.1016/j.pneurobio.2016.01.005

[10] Shigemoto-Mogami Y, Hoshikawa K, Goldman JE, et al. Microglia enhance neurogenesis and oligodendrogenesis in the early postnatal subventricular zone. J Neurosci. 2014;34:2231–2243. 24501362 10.1523/JNEUROSCI.1619-13.2014 PMC3913870

[11] Yang Y, Zhang M, Kang X, et al. Thrombin-induced microglial activation impairs hippocampal neurogenesis and spatial memory ability in mice. Behav Brain Funct. 2015;11:30. 26410080 10.1186/s12993-015-0075-7 PMC4584127

[12] Guan YZ, Sun C, Wang HL, et al. MiR-223-5p inhibitor suppresses microglia inflammation and promotes Nrg-1 in rats of spinal cord injury. Eur Rev Med Pharmacol Sci. 2019;23:9746–9753. 31799641 10.26355/eurrev_201911_19537

[13] Yang YH, Zhu J. Targeting miR-106-3p facilitates functional recovery via inactivating inflammatory microglia and interfering glial scar component deposition after neural injury. Eur Rev Med Pharmacol Sci. 2019;23:9000–9008. 31696488 10.26355/eurrev_201910_19300

[14] Amici SA, Dong J, Guerau-de-Arellano M. Molecular mechanisms modulating the phenotype of macrophages and microglia. Front Immunol. 2017;8:1520. 29176977 10.3389/fimmu.2017.01520 PMC5686097

[15] Varol D, Mildner A, Blank T, et al. Dicer deficiency differentially impacts microglia of the developing and adult brain. Immunity. 2017;46(1030–44.e8):1030–1044.e8. 28636953 10.1016/j.immuni.2017.05.003

[16] Sharma N, Verma R, Kumawat KL, et al. miR-146a suppresses cellular immune response during Japanese encephalitis virus JaOArS982 strain infection in human microglial cells. J Neuroinflammation. 2015;12:30. 25889446 10.1186/s12974-015-0249-0 PMC4355369

[17] Cardoso AL, Guedes JR, Pereira de Almeida L. Pereira de Almeida L, Pedroso de Lima MC. miR-155 modulates microglia-mediated immune response by down-regulating SOCS-1 and promoting cytokine and nitric oxide production. Immunology. 2012;135(1):73–88. 22043967 10.1111/j.1365-2567.2011.03514.x PMC3246654

[18] Nie H, Hu Y, Guo W, et al. miR-331-3p inhibits inflammatory response after intracerebral hemorrhage by directly targeting NLRP6. Biomed Res Int. 2020;2020:6182464. 32596340 10.1155/2020/6182464 PMC7298275

[19] Zou Z-Y, Liu J, Chang C, et al. Biliverdin administration regulates the microRNA-mRNA expressional network associated with neuroprotection in cerebral ischemia reperfusion injury in rats. Int J Mol Med. 2019;43:1356–1372. 30664169 10.3892/ijmm.2019.4064 PMC6365090

[20] Jiang S, Li X, Wang X, et al. MicroRNA profiling of the intestinal tissue of Kazakh sheep after experimental Echinococcus granulosus infection, using a high-throughput approach. Parasite. 2016;23:23. 27235195 10.1051/parasite/2016023 PMC4884269

[21] Ye Z, Hu J, Xu H, et al. Serum Exosomal microRNA-27-3p aggravates cerebral injury and inflammation in patients with acute cerebral infarction by targeting PPARγ. 10.1007/s10753-020-01399-3 33394189

[22] Hongxia L, Yuxiao T, Zhilei S, et al. Zinc inhibited LPS-induced inflammatory responses by upregulating A20 expression in microglia BV2 cells. J Affect Disord. 2019;249:136–142. 30772740 10.1016/j.jad.2019.02.041

[23] Chen L, Fang Z, Wang X, et al. G protein-coupled receptor 39 activation alleviates oxidized low-density lipoprotein-induced macrophage inflammatory response, lipid accumulation and apoptosis by inducing A20 expression. Bioengineered. 2021;12:4070–4080. 34288802 10.1080/21655979.2021.1952917 PMC8806696

[24] Voet S, Mc Guire C, Hagemeyer N, et al. A20 critically controls microglia activation and inhibits inflammasome-dependent neuroinflammation. Nat Commun. 2018;9:2036. 29789522 10.1038/s41467-018-04376-5 PMC5964249

[25] Parisi C, Napoli G, Amadio S, et al. MicroRNA-125b regulates microglia activation and motor neuron death in ALS. Cell Death Differ. 2016;23:531–541. 26794445 10.1038/cdd.2015.153 PMC5072447

[26] Dalal NV, Pranski EL, Tansey MG, et al. RNF11 modulates microglia activation through NF-κB signalling cascade. Neurosci Lett. 2012;528:174–179. 22975135 10.1016/j.neulet.2012.08.060 PMC3478679

[27] Meng Z, Zhao T, Zhou K, et al. A20 Ameliorates Intracerebral Hemorrhage-Induced Inflammatory Injury by Regulating TRAF6 Polyubiquitination. J Immunol. 2017;198(2):820–831 27986908 10.4049/jimmunol.1600334 PMC5220121

[28] Guo Q, Dong H, Liu X, et al. A20 is overexpressed in glioma cells and may serve as a potential therapeutic target. Expert Opin Ther Targets. 2009;13:733–741. 19492975 10.1517/14728220903045018

[29] Gao L, Zhao F, Zhang Y, et al. Diminished ovarian reserve induced by chronic unpredictable stress in C57BL/6 mice. Gynecological Endocrinol. 2020;36:49–54. 10.1080/09513590.2019.1631274 31269828

[30] Regen T, van Rossum D, Scheffel J, et al. CD14 and TRIF govern distinct responsiveness and responses in mouse microglial TLR4 challenges by structural variants of LPS. Brain Behav Immun. 2011;25:957–970. 20951794 10.1016/j.bbi.2010.10.009

[31] Wang X, Li T. Ropivacaine inhibits the proliferation and migration of colorectal cancer cells through ITGB1. Bioengineered. 2021;12:44–53. 33345684 10.1080/21655979.2020.1857120 PMC8806321

[32] Ayyadevara VSSA, Roh K-H. Calcium enhances polyplex-mediated transfection efficiency of plasmid DNA in Jurkat cells. Drug Deliv. 2020;27:805–815. 32489110 10.1080/10717544.2020.1770371 PMC8216448

[33] Sarsour EH, Kalen AL, Xiao Z, et al. Manganese superoxide dismutase regulates a metabolic switch during the mammalian cell cycle. Cancer Res. 2012;72:3807–3816. 22710435 10.1158/0008-5472.CAN-11-1063 PMC3429130

[34] Kopra J, Villarta-Aguilera M, Savolainen M, et al. Constitutive Ret signaling leads to long-lasting expression of amphetamine-induced place conditioning via elevation of mesolimbic dopamine. Neuropharmacology. 2018;128:221–230. 29031851 10.1016/j.neuropharm.2017.10.010 PMC6104196

[35] Chen X, Qian B, Kong X, et al. A20 protects neuronal apoptosis stimulated by lipopolysaccharide-induced microglial exosomes. Neurosci Lett. 2019;712:134480. 31493550 10.1016/j.neulet.2019.134480

[36] Pilipović I, Stojić-Vukanić Z, Prijić I, et al. Propranolol diminished severity of rat EAE by enhancing immunoregulatory/protective properties of spinal cord microglia. Neurobiol Dis. 2020;134:104665. 31689515 10.1016/j.nbd.2019.104665

[37] Ta TT, Dikmen HO, Schilling S, et al. Priming of microglia with IFN-γ slows neuronal gamma oscillations in situ. Proceedings of the National Academy of Sciences of the United States of America 2019; 116(10):4637–4642 30782788 10.1073/pnas.1813562116 PMC6410786

[38] Kolosowska N, Keuters MH, Wojciechowski S, et al. Peripheral administration of IL-13 induces anti-inflammatory microglial/macrophage responses and provides neuroprotection in ischemic stroke. Neurotherapeutics. 2019;16:1304–1319. 31372938 10.1007/s13311-019-00761-0 PMC6985054

[39] Venkatesan C, Chrzaszcz M, Choi N, et al. Chronic upregulation of activated microglia immunoreactive for galectin-3/Mac-2 and nerve growth factor following diffuse axonal injury. J Neuroinflammation. 2010;7:32. 20507613 10.1186/1742-2094-7-32 PMC2891720

[40] Cai H, Liang Q, Ge G. Gypenoside attenuates β amyloid-induced inflammation in N9 Microglial cells via SOCS1 signaling. Neural Plast. 2016;2016:6362707. 27213058 10.1155/2016/6362707 PMC4861811

[41] Oksanen M, Lehtonen S, Jaronen M, et al. Astrocyte alterations in neurodegenerative pathologies and their modeling in human induced pluripotent stem cell platforms. Cell Mol Life Sci. 2019;76:2739–2760. 31016348 10.1007/s00018-019-03111-7 PMC6588647

