# Supplementary material for: MicroRNA miR-27b-3p regulate microglial inflammation response and cell apoptosis by inhibiting A20 (TNF-α-induced protein 3)
Source: Bioengineered. 2021 Dec 13;12(2):9902–13. doi: 10.1080/21655979.2021.1969195 (PMC8810141; doi:10.1080/21655979.2021.1969195)
Supplement: Supplemental Material [file KBIE_A_1969195_SM2028.zip › supp.pdf]

Table S1. Primer sequences used to construct plasmids.

Construct	Direction	Sequence (5' - 3')
LV-A20 overexpression	Forward	ATGGCTGAACAAGTCCTTCC
	Reverse	TTAGCCATACATCTGCTTGAAC
LV-siRNA-A20		GCTATCACTCATGGATATAAA
siRNA-A20 control		TGCATTCGATTTGATAACAAA
miR-27b-3p mimics		UUCACAGUGGCUAAGUUCUGC
mimics control		UUCUUCGAACGUGUCACGUTT
miR-27b-3p inhibitor		GCAGAACUUAGCCACUGUGAA
Inhibitor control		CAGUACUUUUGUGUAGUACAA

Table S2. Primer sequences for real-time RT-PCR.

Gene	Direction	Sequence (5' - 3')
IL-6	Forward	TAGTCCTTCCTACCCCAATTTCC
	Reverse	TTGGTCCTTAGCCACTCCTTC
IL-1β	Forward	TGCCACCTTTTGACAGTGATG
	Reverse	TGATGTGCTGCTGCGAGATT
TNF-α	Forward	CAAGGGACAAGGCTGCCCCG
	Reverse	GCAGGGGCTCTTGACGGCAG
A20	Forward	GACCCTGAAGGACAGTGGAC
	Reverse	TTTGCAGCGTTGATCAGGTG
β-actin	Forward	CGATGGGTTGTACCTTGTCTAC3
	Reverse	GCAGAGAGGAGGTTGACTTTC3
miR-27b-3p	Forward	TTCACAGTGGCTAAG
	Reverse	GTGCAGGGTCCGAGGT
